# Reply to Sikiric et al. BPC 157 Therapy: Targeting Angiogenesis and Nitric Oxide’s Cytotoxic and Damaging Actions, but Maintaining, Promoting, or Recovering Their Essential Protective Functions. Comment on “Józwiak et al. Multifunctionality and Possible Medical Application of the BPC 157 Peptide—Literature and Patent Review. *Pharmaceuticals* 2025, *18*, 185”

**DOI:** 10.3390/ph18101451

**Published:** 2025-09-28

**Authors:** Michalina Józwiak, Marta Bauer, Wojciech Kamysz, Patrycja Kleczkowska

**Affiliations:** 1 Maria Sklodowska-Curie Medical Academy in Warsaw, 03-411 Warsaw, Poland; michalina1395@interia.pl; 2 Department of Analytical Chemistry, Faculty of Pharmacy, Medical University of Gdansk, 80-416 Gdansk, Poland; marta.bauer@gumed.edu.pl; 3 Department of Inorganic Chemistry, Faculty of Pharmacy, Medical University of Gdansk, 80-416 Gdansk, Poland; wojciech.kamysz@gumed.edu.pl; 4 Department of Biomedical Research, National Medicines Institute, 00-725 Warsaw, Poland

We wish to thank the Authors of the comment for their interest in our manuscript [1]. We understand and respect that they defend the peptide they have worked on for many years. However, scientific integrity requires that arguments be based on a comprehensive and balanced body of data—rather than on selectively chosen fragments of the literature.

In the comment [2], the Authors attempt to ignore any concerns regarding the pro-angiogenic and nitric oxide–related activities of BPC 157 by claiming that the peptide merely “modulates” these pathways and therefore cannot cause any harm. However, most of the available studies originate from a single research center (over 80% of all records under “BPC 157” on Google Scholar/Pubmed are linked to P. Sikiric’s group), and experiments routinely employ only a single dose of the compound (10 μg/kg, 10 ng/kg). As a result, there is no crucial information on what occurs with higher, repeated, or long-term exposures—especially given pharmacokinetic data indicating a short plasma half-life (less than 30 min in rats and dogs) and low bioavailability after intramuscular administration [3]. This is especially important as the compound is commercially available as 200 μg capsules (please see: [4]). Moreover, given the rapid plasma clearance, it is critical to investigate whether higher or repeated doses might induce excessive rather than balanced NO release. In this aspect, especially considering that most studies use only one dosage level, this precludes understanding whether BPC-157’s effects are dose-dependent, saturable, or potentially harmful at higher levels. The lack of basic pharmacodynamic and pharmacokinetic profiling is a major gap in the scientific validation of BPC-157.

Importantly, the group of Sikiric et al. in their comment indicated that in the toxicity assessment, BPC 157 was administered at a dose of 2 g/kg, which corresponds to only 46–60 mg per animal for mice weighing 23–30 g. Considering the numerous reports of therapeutic activity at microgram or even nanomolar levels, the complete absence of any observable toxic or behavioral effects at such a high dose is surprising. Intravenous administration bypasses the gastrointestinal tract, yet the lack of effect may reflect rapid systemic clearance, instability, or insufficient peptide content. Overall, this discrepancy calls for clarification regarding the route of administration, compound stability, bioavailability, and the methods used to assess toxicity.

Also, the same authors previously reported increases in VEGF, CD34, and FVIII in the muscle and tendon models, concluding that BPC 157 “stimulates angiogenesis” [5]. In a 2014 review, the same team described VEGF/eNOS activation as a key mechanism of the peptide’s action [6]. Furthermore, an independent group from Taiwan showed that in isolated aortic tissue, BPC 157 disrupts the Cav-1–eNOS inhibitory complex and directly promotes nitric oxide production in a dose-dependent manner [7]. In our opinion, in order to have a strong confirmation of the BPC 157 up- or down-regulatory impact on NO levels, direct measurements of NO, NOx, eNOS phosphorylation, or NOS activity should be performed. The loss or enhancement of the pharmacological effect followed by L-NAME (NOS inhibitor) or L-arginine (substrate) administration should not be interpreted as “proof” of NO involvement. Consequently, several findings clearly indicate that the peptide can powerfully stimulate the NO system at sufficient concentrations; this effect cannot be ruled out at higher clinical doses, which have not yet been studied. Therefore, assertions that “BPC 157 only balances the NO system” remain hypothetical.

As we previously indicated, a PubMed search (20 May 2025) retrieves >190 articles containing “BPC 157”; >80% list P. Sikiric or S. Seiwerth as first- or senior author. Independent laboratories have contributed only a handful of in vitro or short-term rodent studies. Heavy reliance on self-replication inevitably restricts generalizability and increases the risk of confirmation bias.

The comment by Sikiric et al. cites “anti-tumor potential” yet offers no published in vivo tumor-growth or metastasis studies. A single melanoma cell-line experiment from 2004 [8] (unreplicated) is insufficient to support anticancer claims, while pro-angiogenic signaling remains a plausible tumor-promoting hazard. To date, no published in vivo data demonstrate that BPC 157 inhibits tumor progression, reduces tumor volume, or suppresses metastasis.

In line with this, in the comment, the authors claim that oncologic risks are “entirely excluded,” yet they fail to cite a single in vivo study involving solid tumors. Paradoxically, their own previous work demonstrates BPC 157 activates angiogenic signaling—precisely the kind of mechanism known in oncology to potentially support tumor growth. The study by Kang et al. [9], which was cited by the group of Sikirc et al. as a solid confirmation of in vivo anti-tumor activity mediated by BPC peptide, primarily focused on alleviating symptoms of cachexia and inflammation in tumor-bearing animals, rather than inhibiting tumor growth or destroying cancer cells. Therefore, such studies cannot be interpreted as the evidence of BPC 157’s antitumor activity but rather as a potential supportive effect on the overall condition of the diseased organism.

Finally, one of the few registered clinical trials is a Phase I study (NCT02637284) [10], sponsored by PharmaCotherapia, aimed to assess the safety and pharmacokinetics of Bepecin (BPC 157) in healthy volunteers. The plan included single and multiple oral dose administrations in various regimens. However, according to available information, this study was cancelled, and its current status is unknown. Other sources mention earlier clinical studies conducted on patients with multiple types of knee pain [11,12]. However, details of these studies are limited, and their results have not been widely published or independently verified. Also, BPC (known as PL 14736) has been investigated for its potential in treating IBD, particularly ulcerative colitis. A Phase I study assessed its safety, tolerability, and pharmacokinetics in healthy male volunteers. The peptide was found to be safe, but detailed results are scarce [13]. Similarly, Lee and Burgess [14] reported no adverse effects following intravenous infusion of BPC 157 safety (1 to 20 mg) in healthy subjects, though the study included only two participants. Such limited clinical documentation does not meet the criteria for establishing a compound as a safe and effective medicinal product.

Notably, the Authors of the comment report that the peptide used in their experiment was commercially sourced and had a stated purity of 99%. Based on our extensive experience, this appears highly unlikely. Our team, which includes Professor Wojciech Kamysz, a distinguished expert in peptide synthesis and the inventor of numerous peptides and peptide-synthesis equipment, has synthesized several thousand peptides. Achieving 99% purity is exceptionally difficult. Moreover, the available sources do not provide access to key analytical data, including HPLC, ion chromatography (IC), or mass spectrometry (MS) results. It is also unclear what the actual peptide content of the sample was or whether amino acid analysis or elemental analysis was conducted. In addition, there is no information regarding the counterions present in the peptide formulation. Overall, referring to the compound as “commercially available” does not resolve this issue.

We acknowledge the Authors’ passion and long-standing work with BPC 157, and we respect their dedication. However, the science advances through critical verification rather than by repeatedly affirming findings within the same research group. Our publication introduced new hypotheses regarding the potential long-term effects of BPC 157 administration. This does not imply that we deny its beneficial actions—in fact, we acknowledge and respect its therapeutic potential. However, we highlight important areas that warrant further investigation, including observations of possible adverse effects following prolonged exposure to the compound. Such caution is essential for a comprehensive and rigorous assessment of its safety profile. We therefore encourage our colleagues to engage more openly with modern pharmacology, especially with regard to the ambivalent nature of angiogenesis and the complex effects of NO system modulation. Without independent, well-designed multi-dose studies, long-term safety evaluations, and randomized clinical trials, it will remain difficult to convince the broader medical community that this so-called “wonderful” compound meets the criteria of a legitimate therapeutic agent.
